# “Bailout” Endovascular Treatment of Acute Aortic Occlusion

**DOI:** 10.1155/2018/6083802

**Published:** 2018-04-30

**Authors:** Konstantinos Tigkiropoulos, Kyriakos Stavridis, Ioannis Lazaridis, Nikolaos Saratzis

**Affiliations:** Vascular Unit, 1st Department of Surgery, Papageorgiou General Hospital, Aristotle University of Thessaloniki, Thessaloniki, Greece

## Abstract

A 37-year-old man who had a recent history of acute myocardial infarction (AMI) 3 months ago presented to the emergency department with acute ischemia of lower limbs. A CT aortography was performed, where left ventricle thrombi and acute thromboembolic occlusion of aortoiliac bifurcation were depicted. He was urgently transferred to the operation theatre, where Fogarty embolectomy was initially unsuccessful. He was managed by primary deployment of balloon expandable (BE) covered stents in the aortic bifurcation followed by thrombectomy of the left ventricle (LV) under extracorporeal circulation by cardiothoracic surgeons 2 days after initial operation. He was discharged in good general condition after 20 days under warfarin and aspirin therapy.

## 1. Introduction

Cardiovascular diseases are the leading cause of mortality in western societies. Mortality from AMI has decreased after introduction of percutaneous coronary intervention (PCI); nevertheless postinfarct complications are still a main cause of increased morbidity and mortality.

One of the most serious complications is the thromboembolic event from left ventricular thrombi formation causing cerebrovascular accident and visceral and limb ischemia. Incidence of left ventricular thrombus formation after anterior AMI is 5.4% and 7.1% in two studies [1, 2]. We present the case of acute embolic occlusion of aortoiliac bifurcation from left ventricular thrombi in a 37-year-old man treated endovascularly followed by surgical removal of left ventricle thrombi.

## 2. Case Report

A 37-year-old man, whose weight is 105 kg (BMI index: 30,68 Obese class I), presented to the emergency department with complaint of acute onset of bilateral limb pain in the last 3 hours. He was a smoker and had a recent history of AMI 3 months ago treated medically. His medical history included a deep vein thrombosis of left leg after a motor accident 10 years ago.

On clinical examination, lower limbs were painful, cold, and pale with muscle paralysis and anesthesia. Infrainguinal arterial pulses were absent. Cardiac examination showed normal heart sounds with no murmur or gallop.

**Figure 1:**
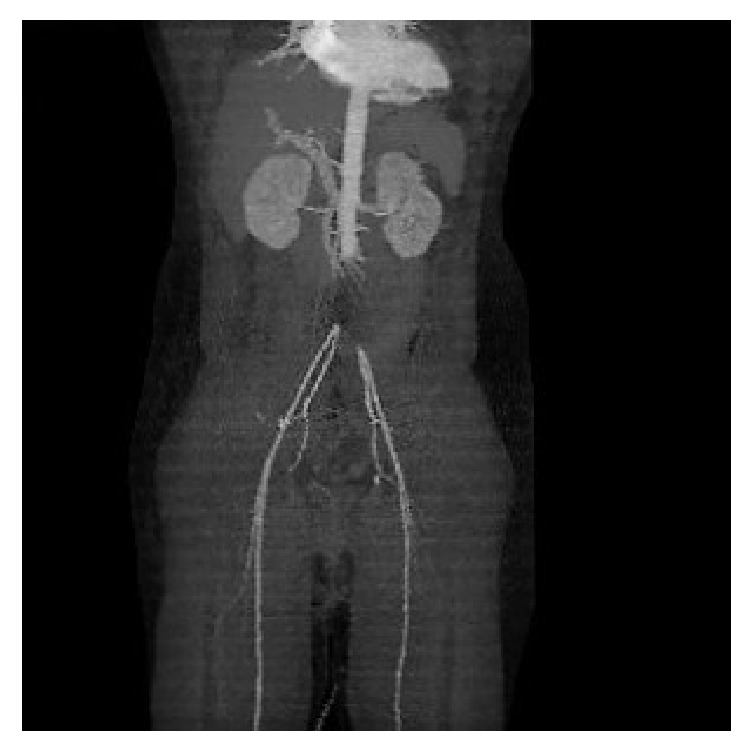
CT angiography depicted occlusion of infrarenal aorta and aortoiliac bifurcation.

Chest X-ray was normal. A provisional diagnosis of acute ischemia of lower limbs was established and 5000 units of unfractionated heparin were given intravenously. The patient performed a thoracoabdominal CT angiography, where occlusion of aortoiliac bifurcation was depicted (Figure 1).

**Figure 2:**
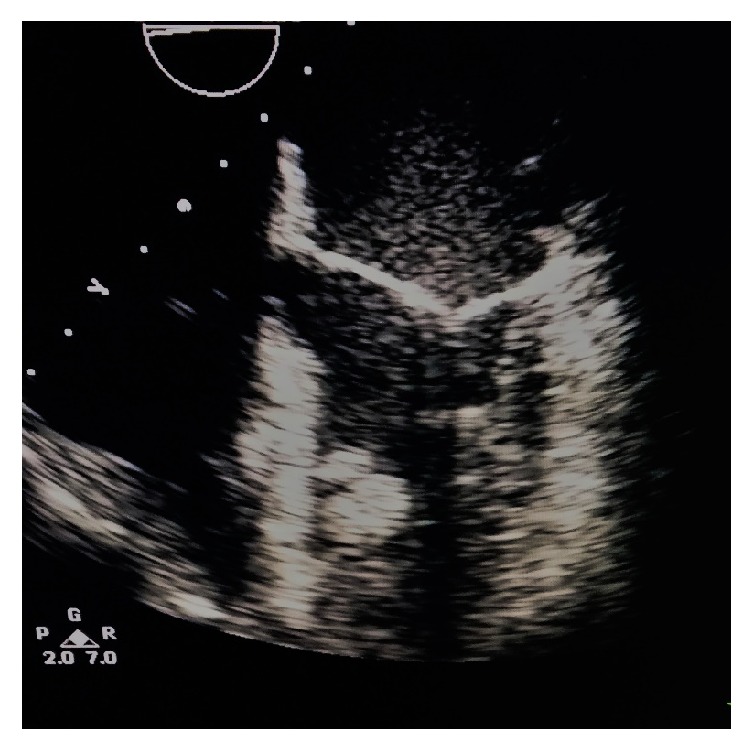
Transesophageal echocardiography showed two pedunculated thrombi in the left ventricle.

Preoperative anesthesiological evaluation was performed, which demanded an emergent cardiological examination due to his recent medical history. A transthoracic echocardiography was performed, which showed two mobile pedunculated thrombi that were approximately 2 cm in diameter in the left ventricle, without regurgitation or stenosis of mitral and aortic valve and ejection fraction, 60% (Figure 2).

## 3. Surgical Treatment

Immediate consult of vascular and cardiothoracic surgery units was scheduled. It was decided that acute occlusion of abdominal aorta would be managed in priority due to established ischemia of lower limbs. The procedure was carried out in a fully equipped operation suite under general anesthesia. Surgical access was gained through common femoral arteries in standard surgical fashion. The patient was systematically heparinized (75 units/kg) and arteriotomies were performed. Fogarty catheters number 5 were advanced to the abdominal aorta with unsuccessful removal of thromboembolic material, which probably was adherent to the aortic wall. Due to his obesity and his medical history, an endovascular treatment was emergently decided as a bailout procedure for revascularization of lower limbs. Two 0.035′′ guidewires (Glidewire, Terumo Corporation, Japan) were successfully advanced proximally to the ascending aorta from each side. A 7-F, 45 cm in length Arrow sheath (Arrow International, Inc., Reading, PA, USA) was introduced over the wire and advanced to the level of abdominal aortic occlusion and aortography was performed.

**Figure 3:**
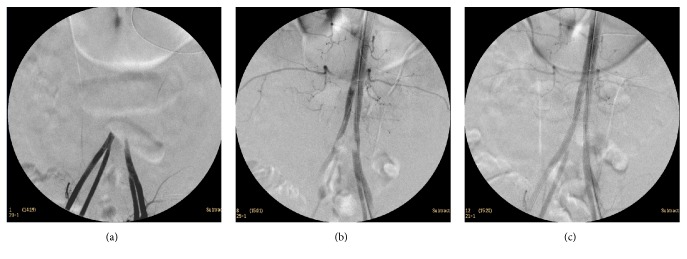
Intraoperative DSA revealed occlusion of aortoiliac bifurcation. Final angiography after deployment of balloon expandable covered stents with excellent runoff without any residual stenosis.

Two balloon expandable (BE) covered stents (Bentley Innomed GmbH, Germany, 10 × 57 mm (right side) and 9 × 57 mm (left side)) were initially advanced and deployed at aortic bifurcation. An aortography was performed, which showed residual thrombus at the distal end of both common iliac arteries. Two balloon expandable covered stents (Bentley Innomed GmbH, Germany, 10 × 37 mm (right side) and 9 × 37 (left side)) were additionally deployed caudally. Final angiography showed patent aortic and iliac bifurcation without stenosis (Figures 3(a)–3(c)). Fasciotomies of anterior and lateral compartments of both legs were subsequently performed to treat compartment syndrome. The patient was transferred to the intensive care unit and he was hemodynamically stable. Aggressive intravenous hydration was administered to protect renal function from reperfusion injury and myoglobinuria. As the potential risk of embolization was considered to be high, in order to prevent further thromboembolic events, surgical thrombectomy was recommended after consultation of intensive care unit and cardiothoracic specialists. Under extracorporeal circulation, surgical thrombectomy of the left ventricle was successfully performed 2 days after primary operation.

The patient had an uneventful postoperative recovery. A thrombophilic screening including antithrombin III, protein C, protein S, anticardiolipin antibodies, Factor V Leiden, and prothrombin gene 202210A mutation was negative for coagulopathy. The patient was started on LMWH (Tinzaparin 18000 units) during his hospitalization. He was discharged in good clinical condition 20 days later under warfarin and antiplatelet therapy (aspirin).

## 4. Follow-Up

**Figure 4:**
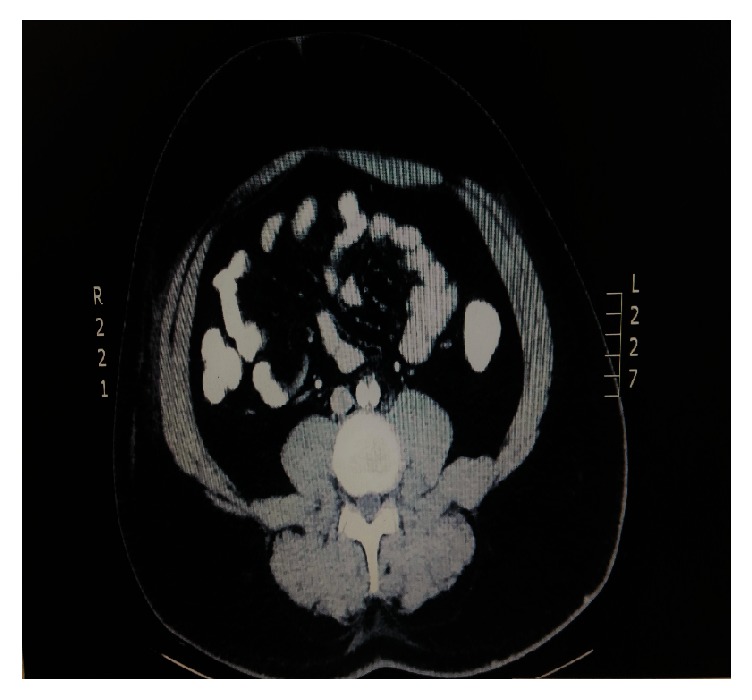
CT aortography depicted excellent patency of covered stents after 1 year.

The patient was followed up according to our department's protocol, which included clinical examination at vascular ward and a CT angiography of abdominal aorta at 6 and 12 months and then annually for at least 3 years. CT aortography depicted patent covered stents with excellent runoff and absence of any residual stenosis (Figure 4).

## 5. Discussion

Acute aortic occlusion is a rare life-threatening condition with increased mortality ranging from 20 to 50%. It occurs usually secondary to thromboembolism, acute dissection, trauma and in situ thrombosis, coagulopathy, and vasculitis [3]. The heart is a major source of emboli in patients with recent myocardial infarction, valvular disease, and atrial fibrillation. One of the most common complications of myocardial infarction is the development of left ventricular thrombus due to hypokinesia of the anterior wall of the heart [4].

When provisional diagnosis of acute aortic occlusion is suspected, imaging of the thoracic and abdominal aorta is necessary to evaluate the source of emboli as well as the extent of the disease. Nowadays, CT angiography has become the imaging modality of choice because it is a noninvasive, less time-consuming technique with high sensitivity and specificity over 90% [5, 6].

Treatment options for acute aortic occlusion include surgical revascularization with Fogarty catheter, direct aortoiliac thrombectomy, catheter-directed thrombolysis, percutaneous techniques (aspiration thrombectomy and mechanical thrombectomy), and endovascular stenting [7]. Thromboembolectomy using Fogarty catheter is the classic surgical treatment of acute arterial occlusion. However, repeated manipulations can cause endothelial injury, local dissection, and distal embolization and a high number of patients (>20%) require secondary procedures [8]. Catheter-directed intra-arterial thrombolysis is a widely accepted technique with clot lysis rate of approximately 70% [7, 8]. Despite this fact, it is not used in patients with critical ischemia because it is a time-consuming process with serious hemorrhagic and distal embolization complications [9]. Percutaneous techniques have been widely used but they are associated with significant blood loss, vascular injury at the access site, and distal embolization as well [9, 10]. Endovascular stenting with covered stents is not considered as a first-line treatment of acute arterial occlusion and is associated with high rate of distal embolization. Nevertheless, there are some advantages over other techniques. It is not a time-consuming technique. This is important in therapy of ischemia-reperfusion injury, which is the main cause of morbidity and mortality in these patients. Rapid restoration of aortic patency with deployment of stent grafts without use of aortic clamp is an additional factor, providing hemodynamic stability, avoiding the stress of aortic cross-clamping. Additionally, deployment of covered stents across occlusion is not technically demanded and the profile of delivery system is small (7 Fr), reducing vascular access site injuries [11]. It may be used as a bailout procedure in cases where open surgical procedure or thrombolysis has failed or is not indicated in high-risk patients.

Berczi et al. successfully managed seven acute thrombotic occlusions in the iliac arteries by primary stent implantation without distal embolization [12], whereas Yilmaz et al. treated six embolic occlusions in the iliac arteries by primary stent deployment without complications [13].

Our patient had a recent history of myocardial infarction with left ventricular thrombus embolized in the aortoiliac bifurcation. High index of suspicion and immediate treatment are crucial for reperfusion of lower limbs. Direct aortoiliac thrombectomy was not performed due to his obesity and his recent medical history. Primary endovascular stenting of aortic bifurcation, a minimal invasive technique, was the treatment of choice without any complication and excellent patency after 12 months.

Pedunculated mobile thrombi of left ventricle have high rate of systemic embolization. Definite therapy has not yet been established. Nowadays, first line of treatment for a LV thrombus is anticoagulation. Oral anticoagulants have had variable success, with resolution rates ranging from 13 to 59% [14]. Thrombolysis with urokinase and tissue plasminogen activator has been reported, but the risks of hemorrhagic or embolic complications may be high [15]. However, large free-floating thrombus as is in this case often requires urgent surgical thrombectomy

In conclusion, acute aortic occlusion is a serious life-threatening event with devastating complications if left untreated or if the treatment was delayed. Endovascular stenting may be used as a bailout procedure in cases where surgical embolectomy and thrombolysis are unsuccessful. Despite encouraging results, further studies are necessary to establish its efficacy.
